# Effect of Horticultural Mineral Oil on Huanglongbing Transmission by *Diaphorina citri* Kuwayama (Hemiptera: Psyllidae) Population in a Commercial Citrus Orchard in Sarawak, Malaysia, Northern Borneo

**DOI:** 10.3390/insects12090772

**Published:** 2021-08-28

**Authors:** Sui S. Leong, Stephen C. T. Leong, George Andrew Charles Beattie

**Affiliations:** 1 Faculty of Agricultural Science and Forestry, Universiti Putra Malaysia Campus Bintulu Sarawak, Nyabau Road, Bintulu 97008, Sarawak, Malaysia; leongchanteck@gmail.com; 2 Centre for Plant and Food Science, University of Western Sydney, Locked Bag 1797, Penrith, NSW 2751, Australia; A.Beattie@westernsydney.edu.au

**Keywords:** *Diaphorina citri*, Huanglongbing disease, *Rutaceae*, flush shoot, horticultural mineral oil

## Abstract

**Simple Summary:**

Increase in abundance of the vector *Diaphorina citri* Kuwayama population is mostly influenced by the cyclic flushing of citrus. Relative degree of aggregation index for *D. citri* adults increased during periods of cyclic production of new flush as females aggregated to lay eggs, and the adult migration and dispersal was influenced by flushing cycles. Horticultural mineral oil (HMO) can reduce the spread of HLB as HMO produced significantly lower level of 11.43% of diseased citrus trees compared to 42.20% in untreated control plots.

**Abstract:**

*Diaphorina citri* Kuwayama transmits a destructive citrus disease caused by a fastidious bacterium ‘*Candidatus* Liberibacter asiaticus’ (CLas) designated as Huanglongbing (HLB) which posed a risk of detrimental threat to the Malaysian citrus industry. All *D. citri* life stages show a lumped habit on young flushes and its population fluctuations was closely related to accessibility of young flushes. The study aimed to investigate if the appearance of young flush shoots on citrus influences ACP population fluctuation and if horticultural mineral oil (HMO) could reduce spread of HLB transmission by ACP in a commercial healthy orchard. Field research was carried out from 1 April 2011 to 1 December 2014 in a 2-year-old 1 ha citrus farm that consisted of 200 PCR-certified disease-free grafted non-bearing honey tangerine (*Citrus reticulata* L.) in southwestern Sarawak, Malaysia. The experiment had two treatments namely control (unsprayed) and nC24 HMO with four replications arranged in a simple randomized block design. ACP eggs, nymphs, and adults per flush shoot was assessed and HLB incidence was monitored for visual inspection of the citrus trees for the current existence of usual signs of characteristic symptoms of HLB such as yellowing shoots, leaf mottling, and corky or enlarged veins on leaves. HLB-specific primer was employed in 16S rDNA polymerase chain reaction to detect the CLas gene in diseased trees. Increase in abundance of *D. citri* is mainly affected by the citrus flushing cycles and their life stages are completed on these flush shoots. Relative degree of aggregation index for *D. citri* adults increased during periods of cyclic production of new flush. HMO-treated plots produced a significantly lower percentage up to 11.43% of diseased trees against 42.20% in untreated control plots. HMO is effective against *D. citri* and recommended to be incorporated in the IPM program to prevent infection and reduce the spread of HLB.

## 1. Introduction

The Asian citrus psyllid (ACP), *Diaphorina citri* (Insecta: Hemiptera: Psyllidae), is an economically important pest insect of citrus as it is one of the two known effective vectors of a bacterium called Huanglongbing (HLB) disease, also designated as citrus greening [1,2,3]. HLB, being one of the ultimate devastating and fastidious citrus diseases produced by ‘*Candidatus* Liberibacter asiaticus’ (CLas) (α-Proteobacteria), is a phloem-limited Gram-negative bacterium [4] attacking sweet orange and mandarin varieties in Asia [5]. It has caused the majority of economic loss in the citrus blossoming in Asia [6], South Africa, and USA [7]. Currently, HLB has ruined citrus farms with a total area of 1143 ha in Sarawak, Malaysia in 1992 as well as the predicted profit reduction of 310,000 trees producing 6500 metric tons of fruit and the commercial loss of Ringgit Malaysia (RM) 6.5 million or USD 1.6 million [8]. According to Beattie and Holford [9], any detection of HLB disease in the year after 1970 would pose a devastating menace to the Malaysian citrus industry. Any effective integrated pest management strategies for *D. citri* needs some knowledge of their dispersion patterns of population distribution between trees within groves. Vegetative growth in citrus trees is affected by environmental factors and occurs in several cycles (flushes) over the year. It is well documented that under Mediterranean and temperate climatic conditions, flushes emerge in well-defined cycles, whereas in tropical and subtropical regions, they appear more unevenly [10]. Despite homogeneity of citrus trees, all life stages of *D. citri* show an aggregated habit on young flushes, where oviposition, and growth of immature nymphs take place [11]. *D. citri* adults usually move between orchards [12]. Although, information on growth of *D. citri* in connection with the development of young citrus flush growth has not been clearly illustrated but it is important for deciding the timing and frequency of control measures required to reduce *D. citri* populations and spread of HLB in orchards [13]. Although eggs, nymphs or adults of *D. citri* can possibly be found on reproductive flushes, the main sites for psyllid development apparently are vegetative flushes. This describes a strong relationship between vegetative flush development and the dynamics of *D. citri* populations in citrus orchards [14,15,16]. *D. citri* is attracted to flushes that provide most favorable conditions for feeding and oviposition, particularly those at the very early stage of development, by a combination of chemical volatiles and visual stimuli [17,18,19,20,21,22]. Husain and Nath [23] reported that oviposition takes place almost entirely on very immature flushes. The time of extensive activity of *D. citri* and most rapid increase in their numbers correlates with that of the growing of new shoots and the emergence of new leaves [23]. Other authors have published that females oviposit their eggs in the growing tips of young flush shoots, choosing flush growth length <6 mm [24,25,26,27] and that nearly all eggs are oviposited within 14 days of new sprouting beginning [24]. Climate, particularly temperature, can influence the development of *D. citri* populations (all life stages) in citrus orchards [28,29,30].

The use of citrus flushing and ACP monitoring tools is essential for the knowledge of the occurrence pattern of cycles of both, and for the improvement of the ACP management. Insecticidal control for *D. citri* is the primary strategy for managing HLB as it is capable to control *D. citri* populations and can significantly reduce CLas transmission rate [31]. However, the application of pesticides in some orchards attempting at eradicating and containing *D. citri* population were unsuccessful and may influence the efficacy of biocontrol agents. Consequently, the development of a more effective biocontrol program for this pest will increase the efficacy and sustainability of other control strategies, including insecticide-reliant programs. Decreased *D. citri* populations developing from natural enemy activity may also control rates of CLas spread and minimize economic losses resulting from HLB spread in orchards. However, it is unlikely to provide complete prevention of CLas spread if control of *D. citri* population is by natural enemies alone [32,33]. Application of selective insecticides [34,35,36,37] to be supplemented by biological control agents such as *Tamarixia radiata* (Waterston) (Hymenoptera: Eupholidae) and *Diaphorencyrtus aligarhensis* (Shafee, Alam and Argarwal) (Hymenoptera: Encyrtidae) as well as several ladybeetle species and Argentine ant *Linepithema humile* (Hymenoptera: Formicidae) are recommended for development of integrated pest and disease management strategy of *D. citri* [38,39,40,41,42,43,44,45]. There is usually no treatment for HLB and the best curative method is to carry out the integrated disease management utilizing planting materials that are healthy and Polymerase Chain Reaction (PCR) certified HLB-free, elimination of symptomatic citrus trees to reduce inoculum sources, monitoring of vector and disease incidence and insecticidal applications [6,7,8], as to reduce the spread of the HLB disease. Preventing the existence of high densities of vector population especially on immature young flush shoots for egg laying is very important for citrus farmers [46]. Ko [47] reported that insecticidal sprays for control of *D. citri* required at least fortnightly, and more periodic applications were needed throughout the span of flushing cycle in Malaysian citrus orchards. A yearly application of 8–18 sprays to trees below 4-years-old is a management strategy currently practised in Brazil [48]. The emerging insecticide resistance problem was reported by Tiwari et al. [7] for *D. citri* if efficacious resistance management is not carried out. ACP can transmit CLas while feeding on citrus flush shoots. Our research linked the phenology of citrus young flush shoots with the development of *D. citri* life stages from egg-laying to development of adult. The objectives of this study aimed to ascertain if appearance of young flush shoots on citrus influences ACP population fluctuation and if horticultural mineral oil (HMO) could reduce the spread of HLB transmission by ACP in a healthy orchard.

## 2. Materials and Methods

### 2.1. Experimental Sites

Field studies was carried out from 1 April 2011 to December 2014 in a 2-years-old (planted in 2009) 1 ha commercial citrus orchard that consisted of 200 PCR-certified disease-free grafted non-bearing honey tangerine (*C. reticulata* Linnaeus) seedlings in 20 rows (N-S) in 6–8 columns at 2.0 m within-row spacing located in southwestern Sarawak in Malaysia. The citrus trees of similar size matured rapidly and attained the height of 2–3 m and provided with open canopies at the beginning of the study. A simple complete block (SCB) design was employed to have two treatments with four replications of each treatment in the experiment. A total of 8 plots were used in this experiment. An experimental plot had 20 or more trees. Each replicate of a treatment comprised of 20 trees and 10 to 16 trees in a single row were randomly sampled; evaluations were determined from 10 inner trees in each replicate. Other trees in each plot were used as control guard row to keep away from spray drift in between plots.

The two treatments were listed as follows:

- A control, unsprayed,
- nC24 HMO (Ampol D-C) Tron plus: Ampol Rural, Sydney, Australia, applied at concentrations of 0.33–0.5% (*v/v*), mostly 0.5%.

All trees were sprayed with the fungicide mancozeb (Dithane M45 80 WP) at 3 g product/L of water for protective action against mite/scale infestation and greasy spot when necessary. So far, no phytotoxicity has been observed in the plant, especially on fruit. The guidelines of the HMO were given by Rae et al. [49]. Spray volume per tree became greater while the tree increased in size. Each tree was applied with 3–5 L of spray fortnightly throughout the study with a motorized Fuji FP-25 knapsack sprayer.

### 2.2. Sampling

In this study, 100 random samples of young flush shoot (immature with expanded and tender leaves) that were chosen as the sampling unit were collected weekly. Each flush was around 10–15 mm in length with five chosen young leaves. Count of each life stage such as eggs, nymphs, and adults appearing on each flush shoot on the five twigs (each 10 to 20 cm long) per tree were carried out with the help of a 10× hand lens. Numbers of adults were counted and recorded first, and then the numbers of eggs and nymphs were counted in situ using a 10x hand lens per flush and recorded. Extent of percentage infestation of flush shoot was determined as the number of flush shoots infested with any *D. citri* life stage divided by the total number of flush shoots sampled per tree for every sampling duration. Only some proportion of 50 flush shoots invaded with eggs per treatment were reported since counting and determining the exact number of *D. citri* eggs on flush shoots was extremely laborious.

The study of the flushing pattern of citrus at the study site in the citrus orchard that housed 200 citrus trees was carried out from October 2012 to June 2013. Each of the flush points surrounded by the 1 square feet metal frame were considered suitable for study of *D. citri* development. Each of the young flush points were denoted as percent flushing.

### 2.3. Incidence and Transmission of HBL Disease Relating to D. citri Population

HLB disease incidence was monitored and the number of symptomatic trees was quantified from January 2012 to October 2014 for visual inspection of the tree canopies for the indications of typical features of HLB such as yellow shoots, leaf mottling, and corky or enlarged veins on leaves [4]. These are often the first symptoms to appear, followed by small and upright leaves with chloratic patterns which look like those produced by iron and zinc micronutrient deficiencies with twig sectorial dieback, unequal coloration of the fruits and uneven fruit with abandoned seeds. Bove and Da Graca reported that fruit becomes unmarketable and tree death eventually comes about as the disease advanced and became more severe [4,50]. Since disorders such as micronutrient deficiencies (mostly iron, zinc, manganese, and magnesium), physical trunk and root injury, *Phytophthora* root and stem rot, *Citrus tristeza virus* infection, or nutrient toxicity can imitate HLB-related leaves symptoms [4,51]. Samples of leaf tissue showing symptoms gathered from suspect trees were checked for the existence of bacterium CLas using PCR [52].

#### 2.3.1. Diagnostic Protocol for the Causal Agent of Huanglongbing Disease ‘*Candidatus* Liberibacter asiaticus’ (CLas)

Twenty samples comprise of short branches (10–20 cm) with the attached leaves per replicate taken monthly. Leaf/twig samples collected from citrus plants were stored into a sealable plastic bag and the sample was kept cool under the shady places and used for DNA extraction; 80% ethanol or 1% available chlorine was used for surface sterilizing of leaves. The leaf materials were immersed for two minutes, then washed in water and pressing it dry on tissue paper. A sharp knife was used to chop about 1 g fresh weight of middle ribs and/or leaf stalks from leaves and later were chopped into approximately 2 mm lengths. Sub-samples (approximately 100 mg) were taken from the composites of midribs and petioles’ leaf tissue from four leaves for total DNA preparations.

#### 2.3.2. PCR Assays

The number of citrus trees suspected to be infected with HLB in the orchard was determined according to the visible features. The prevalence of the disease was decided as the number of trees showing typical symptom expression divided by total number of trees in the orchard. The number of trees in the orchard exhibiting characteristic symptom expression was computed once a month between 2011 and 2014. HLB infection incidence was computed as the number of trees exhibiting visual symptom expression divided by the total number of trees in the citrus orchard. Detection of ‘*Candidatus* Liberibacter asiaticus’ (CLas) was performed by conventional PCR from leaf midrib. Leaf middle rib samples were gathered monthly on probably affected trees, and field verification was subsequently carried out to confirm on the diseased trees. HLB-specific primer was employed in 16S rDNA polymerase chain reaction as a molecular diagnostic technique [52] to detect the CLas gene in diseased trees.

Positive PCR outcomes for the CLas gene in *D. citri* adults may give suggestive information about the area probably to be subjected to HLB disease invasion, but in present study, the sampled psyllid adults collected from the citrus orchard were not sent for testing in DNA extraction.

### 2.4. Statistical Analysis

A repeated measure analysis of variance (ANOVA) via SAS [53] was employed to determine the impact of spray treatment, block in the study area and sampling period and their interactivities on *D. citri* population numbers, and HLB disease infection extent. The dependent variables in the field experiments were average number of eggs, nymphs, and adults per flush shoot. Quantities of *D. citri* life stages per flush shoot were log(x + 1)-transformed and percentage HLB disease infection extents were arcsine square root transformed before statistical analysis was carried out [54]. Data were transformed to either logarithmic or arcsine square root when required before analysis to normalize the data and stabilize error variances. Mean numbers of *D. citri* for each development stage per flush shoot were calculated over sampling periods and percentage of HLB disease incidence on treated and control citrus plots was compared using SAS 9.1 [53].

## 3. Results and Discussion

### 3.1. Seasonal Variation in D. citri Population Abundance Relating to Flushing

**Figure 1:**
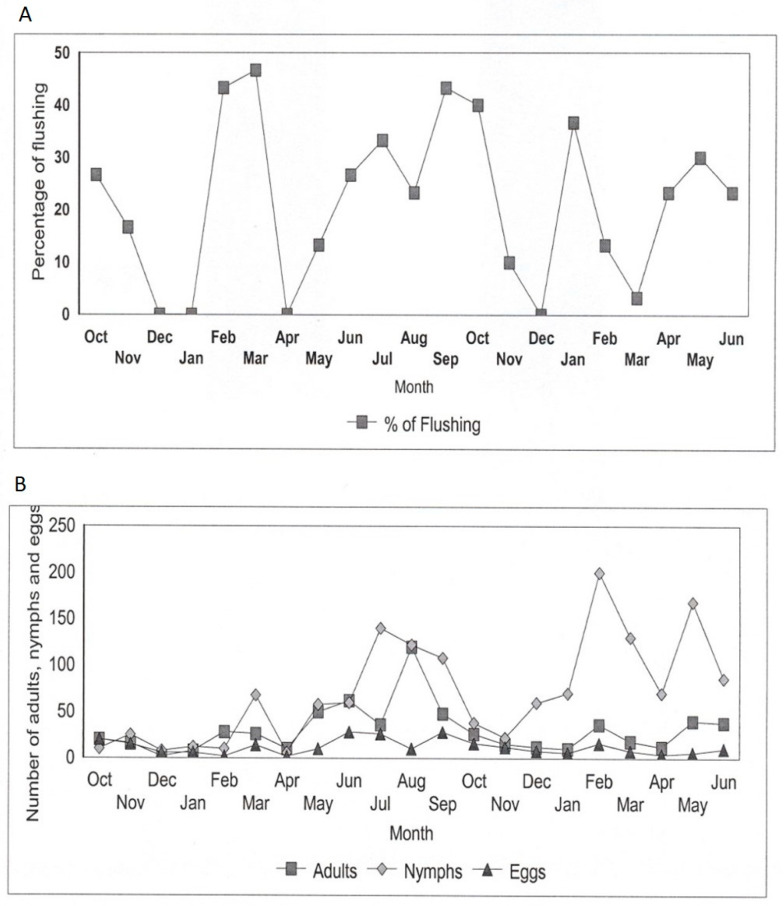
Seasonal variation in *D. citri* population abundance relating to flushing cycle from October 2012 to June 2013 in a citrus orchard; (**A**) Percentages of flushing; (**B**) Numbers of adults, nymphs, and eggs.

Numbers of all development stages of *Diaphorina citri* differed with time of the year, mainly resurging during flushing rhythms when immature fresh flushes appeared (Figure 1). Two to three major flushes were observed during February–March, June–July and September–October throughout study period (Figure 1). There were two to three adult peaks, three nymph peaks and two to three egg peaks annually. *Diaphorina citri* numbers grew throughout flush rhythms whenever new flushing shoots were present and decreased afterwards. There was continuous increased flush infestation levels by ACP from February to September–October whenever highest infestation levels were noticed. The main upsurge in *D. citri* numbers occurred during the main September–October flush cycle which followed the first rain of the rainy season. Moderate populations were observed during the June–July and February–March flush cycles. Numbers of eggs and adults observed on young flushes throughout the dry month flush cycles in June–July were smaller than that noticed throughout different flush cycles despite the presence of nymph population comparable to September–October flush cycle. Such relatively lower densities of *D. citri* egg and adult per flush shoot could be owing to the hot weather prevalent in southwestern Sarawak, Malaysia, between June and August. Leong et al. [28] reported that maximum temperature and relative humidity were negatively correlated with the adult and egg populations. During this hot and dry period of time (June–August), the mean monthly temperature, rainfall and relative humidity is ranging from 29.3 to 33.9 °C, 197 to 207 mm, between 78.7 and 83.35%, respectively. This temperature range is much greater than the ideal temperature of 29.6 °C and 24–28 °C for ACP egg-laying and for growth, respectively [30,54,55,56]. Laboratory studies indicated that *D. citri* population rears optimally at 28 °C in terms of the net reproductive rate and intrinsic rate of increase [55]. Hot temperature may limit movement of ACP and/or decrease its oviposition capability. During dry season, citrus immature flushes also prone to full-grown more quickly owing to warm temperature, therefore the favorable occasion for *D. citri* oviposition and/or may reduce the suitability of flush shoot for development of immature *D. citri*. Milosavljevic et al. [30] who synthesizes datasets of five globally distributed *D. citri* populations (Brazil, California, China, Florida, and Japan) reared on six different host plants reported that daily thermal fluctuations had significant effects on growth times of *D. citri* nymphs, which differed across experimental temperatures. *D. citri* nymphs reared at constant temperatures completed development faster than those reared under fluctuating profiles with comparable temperature means. Psyllid populations increased slowly from May onwards to August during major flushing durations and declined thereafter. From November onwards both adults and nymphs began to increase gradually and reached peak population in February.

The present study clearly indicated that larger numbers of settling *D. citri* adults remained alive when attracted to flushes on citrus trees mostly those at the very initial development stage can provide most favorable conditions for oviposition and feeding. The higher numbers of each ACP development phases were noted throughout September–October flush cycles compared to the February–March and June–July flush cycles conforming to research finding of Sétamou and Bartels [56]. In Malaysia, young flushes are mainly found throughout the distinct flush rhythms occurring between dry season (April–September) and early raining season (October–March) which frequently associate with seasonal peaks of *D. citri* number. It has been demonstrated that *D. citri* numbes on citrus is firmly related to the young flush shoots present on such host plants [11,13]. The biology of reproductive system of *D. citri* is intimately linked to the appearance and growth of flushes, while feather leaves of flush shoots and comparatively young leaves are needed for oviposition and the growth of immature stages namely nymphs, respectively [23]. The present study corroborates the early research finding by Husain and Nath [23] who reported that the time of important activity and most speedy increase in numbers of *D. citri* correlates with those of the development of new flush growths and the emergence of new foliage. Our results corroborate such findings, particularly with respect to Milosavljevic et al. [45] that cumulative temporal *D. citri* populations (i.e., all life stages) in urban areas of southern California frequently correlated with citrus leaf flushing patterns over time and across different climates. Some researchers have revealed that eggs laid by *D. citri* gravids are only in the buds of young flush shoots and nearly all eggs are oviposited in 14 days in the beginning of new bud [24]. Length of fresh growth of leaves <6 mm is preferred and an increase in the flush length will rapidly decrease densities of deposited eggs on a young flush [24,25,26,27].

However, *D. citri* reproduction on citrus were linked to flushing pattern as *D. citri* adults lay eggs completely on newly emerged young flush shoots during which their immatures particularly those nymphs can feed more favorably and finish their life cycle on them. Findings of the current study imply that surveillance activities for detecting potential infestations of *D. citri* should commence at the emergence of the new flush growth in the earlier season as the temperature begins to increase from April onwards. Although, the most conducive environment for increasing occurrence of immatures with regards to eggs and adults was the slow increase in diurnal atmosphere temperatures that gave rise to highest activities for nymph throughout warm and dry month from May to August that also exhibit maximum temperatures to exert considerable impact on nymph number. The prevalence of warmer temperatures can reduce the time required for *D. citri* to accomplish its development stages; this may lead to additional reproductions being given throughout the developing period. Aurambout et al. [57] reported that decrease in availability of young flush growth can exhibit negative effect on the fecundity of gravid psyllid females, this will lead to the production of fewer clutch of eggs.

Our results appeared to corroborate the significance of young flush growth to *D. citri* that has been illustrated by many investigation workers [58,59,60,61,62,63,64]. Cifuentes-Arenas et al. [13] showed that biotic potential of *Diaphorina citri* is not a matter of the length or the age of the flushes (days after budbreak), but the growth stage of flushes within its development. According to Catling [46], ACP adults have a high biological capacity to reproduce offspring with a short life cycle and are therefore allowed to proliferate and exploit their citrus host plants. The prolonged and sustained supply of young citrus flush growth are very attractive to the *D. citri* vector. It is likely that ACP has been co-existed with its host plants in *Rutaceae* family to schedule exactly as to correlate its breeding, egg-laying, egg maturity and juvenile development with the emergence of new flushing shoots.

### 3.2. Influence of Flushing Cycle on the Spatial Dispersion of D. citri Adults

**Figure 2:**
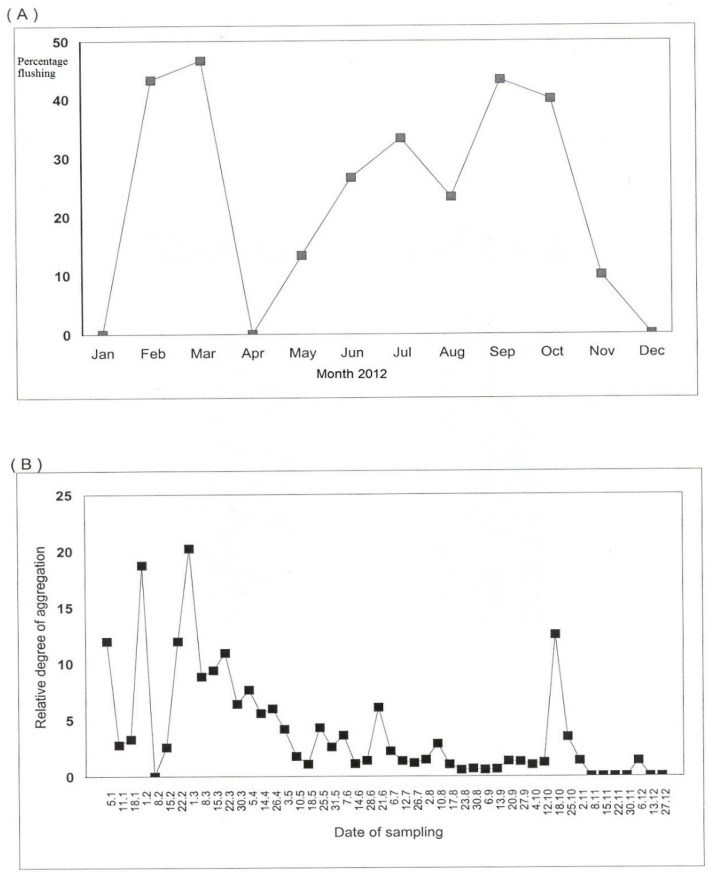
Percentage flushing (**A**) and relative degree of aggregation of *D. citri* adults (**B**) during 2012 on citrus trees.

Numbers of *D. citri* adult differed with time of the year and mainly found throughout flushing rhythms when immature flushing shoots were observed (Figure 2). Three major flushes were produced between February–March, June–July and September–October throughout the study period (Figure 2). The relative degree of aggregation (RDA) index increased during cyclic production of new flush growth and declined thereafter, as it was on these occasions that adult females aggregated on new shoots to lay eggs (Figure 2). Higher numbers of adult *D. citri* observed on new flushes may probably be the consequence of larger numbers of adults come either from the same tree or other trees in the orchard being attracted and settled on citrus orchards. These adult psyllids will probably be attracted to the emergence of new young flush growth and trigger egg-laying while trees are having abundant flushes as exhibited by the higher numbers of egg clutches and immature nymphs on these trees, therefore clarifying the largest population numbers of all development stages noticed on those trees.

During 2012, RDA occurred in early January–February, May–June and October which were coincided with flushing periods. The study showed that the overall population fluctuations of *D. citri* breeding on citrus were influenced by the flushing rhythm (r = 0.58, df = 20; *p* = 0.05). The higher RDA index of *D. citri* adults was observed during the February–March and September–October flush cycles compared to the June–July flush cycle. Therefore, the build-up of the higher adult number occurred in response to flush cycles. The main resources for *D. citri* egg-laying and juvenile development are new flush shoots [55,64] and therefore *D. citri* population fluctuations are closely linked to availability of new flush shoots. The emergence of a new flush cycle initiates a surge in adult number of *D. citri* and a drop was noted while flush shoots reach maturity, and availability of young flushing shoots are reduced in the citrus orchard. Such dynamics in ACP population together with tree phenology certainly specify attraction by immigrating adult psyllids into the citrus trees in orchard during the onset of a flush rhythm. The present investigation corroborates the research finding by Yasuda et al. [63] that *D. citri* prefer young twig of rutaceous host species and their juvenile development completes exclusively on young shoots that strongly monitor dynamics of *D. citri* population in citrus orchards [54,62]. Sétamou et al. [62] reported that *D. citri* adult preferred more favorably young flushing shoots to mature shoots for oviposition, and densities of immature development were certainly associated with tissue softness of flush shoot due to the higher nutritional value of these shoots comparative to mature shoots. Conversely, mature flush shoots with tissue hardness and lower nutritional value may restrict *D. citri* female’s oviposition and immature development. The RDA index reached its highest point in each flush cycle probably when adult eclosion peaked, and then declined gradually with the emigration and dispersal of adults. In 2012, the RDA indicated on flush shoots throughout the rainy month flush rhythms in January–February and October were higher than those observed during March to September in the dry months. Such relatively higher RDA index during January–February and October flush cycles may be due to the high monthly rainfall between 581–919 mm as compared with 374–458 mm from March to September and mean monthly temperature ranging from 22.7 to 29.3 °C during this wet season. Rainfall has been associated with an increase in the *D. citri* population growing on *M. paniculata* by increasing the availability of flush shoots [65]. In addition, this range of temperature is favorable for *D. citri* oviposition and development [54,55,65]. Martini et al. [64] reported that females are stronger fliers than males, dispersal of *D. citri* surges directly within temperature range of ca. 18–33 °C and independent of the relative humidity level. Zorzenon et al. [65] further reported that maximum temperature together with both daily minimum temperature and precipitation in the previous weeks were also associated with production of new citrus flush, which was positively linked to abundance of immigrating *D. citri*.

Dispersal of *D. citri* adult populations and settlement on new citrus trees in the groves was greater during flushing intervals in February–March and September–October (Figure 2). Both *D. citri* adults and nymphs while feeding on citrus flushing shoots can transmit the bacterium CLas. Since there is an intimate relationship among *D. citri* and flush shoots, it is likely that HLB dissemination rates may possibly be affected by the appearance or absence of flushing shoots. Cifuentes-Arenas et al. [13] reported that flush shoots at emerging and growing stages serves as the basis for any biological or chemical control program, to minimize biotic potential of *D. citri* and to protect citrus tree from CLas infection and to minimize HLB transmission. Sétamou et al. [62] further reported that important factors managing citrus host species settlement and behavior of *D. citri* are physical traits and nutrient constituents of flush shoots as well as their nutrient-rich diets, and this interrelation may influence the fluctuations and dissemination of HLB disease in citrus orchards. This may be important in spreading the disease by leading to seasonal migratory as well as dispersal flights and initiation of new infestations to the nearby healthy trees within the citrus orchard. Therefore, the spread of disease seems to be correlated to high infective *D. citri* populations when new flush growths are available. The results were consistent with the psyllid’s need for flush growth to be present for oviposition and nymphal development; a condition undoubtedly lending to contagion of adults coincided with the flush cycles (Figure 2). The substantial and prolonged flow of young flushing citrus trees in February–March and June–October 2012 made them very attractive to ACP. This validates the aggregated character of vector population attracted to the flush cycles of young trees in the field as described by Catling [46]. Supplementary research described by Aubert and Xia [66] suggested that when *D. citri* adult populations attained their top magnitudes in the dry or wet periods, their periodic migrating flights over canopies of *Murraya paniculate (L.)* (Jack) (Plantae: *Rutaceae*) were linked to pressures as a result of congestion of *D. citri* densities. Other factors that also affect the movement or dispersal of *D. citri* are temperature, photoperiod, light intensity, and wind speed [1,6,28,29,30,66].

### 3.3. Effect of Horticultural Mineral Oil (HMO) on Oviposition, Nymphs, and Adult Mortality

Numbers of all *D. citri* life stages and the percent of flushing shoots invaded by its development stages on citrus differed significantly (*F* = 1608.15, df = 1, *p* < 0.001; *F* = 352.11, df = 4, *p* < 0.001) with treatment, sampling time and the treatment by sampling time interrelation (Table 1). Untreated flush accommodated significantly larger numbers of ACP adults, nymphs, and eggs than HMO-treated flush shoots. Similarly, significantly additional untreated flushing shoots were invaded by *D. citri* adults and eggs than HMO- treated flush shoots.

The unsprayed control treatment had significantly (*p* < 0.05) higher mean numbers of adults and nymphs per flush than HMO treatment trees (Table 1). We observe no significant variation between treatments for the number of eggs oviposited per flush, even if extra eggs were oviposited on unsprayed flush shoots (Table 1). The unsprayed control plots had significantly (*p* < 0.05) more flush shoots infested by female psyllids than HMO treatment trees (*p* = 0.01) (Table 1). For eggs, a considerable difference was observed between treatments (*p* = 0.01) for the percent of flush shoot where clutch of eggs was oviposited; HMO treatment importantly decreased the number of flushes where clutch of eggs oviposited. The HMO treatment was effective for reducing the infestation by female psyllids on sprayed flushes. This result may have valuable indications for HLB disease transmission, while it recommends that in this treatment, the range and level of adults feeding might become importantly lower than the unsprayed control treatment, and thus HLB disease transmission would have been comparatively less since the percent of flush shoot infestation by *D. citri* females and percent of flush shoots where clutch of eggs was produced in the HMO treatment was significantly lower compared to the unsprayed treatments (Table 1). Either sprayed or unsprayed flushes have an equal chance to be infested and laid eggs on by female psyllids. However, the deposition of fewer clutches of eggs on sprayed flushes suggests that flush shoots sprayed with HMO were prejudiced against them. This investigation evidently reveals that HMO decreased the number of clutches of eggs laid by gravid females, and are probably the outcome of egg-laying deterrence against *D. citri* female psyllids. Oviposition deterrence activity against gravid females because of oil deposits has also been illustrated with ACP [67,68,69,70], and citrus leaf miner [71,72]. The effect of the HMO on numbers of *D. citri* life stages (see Table 1) was attributed to suffocation of nymphs as well as eggs, and decreased oviposition due to behavioral impacts on gravid females and insecticidal applications can also have resulted in significant adult fatality.

### 3.4. Impact of Horticultural Mineral Oil (HMO) on Disease Incidence and Spread of HBL by D. citri

The percent of HLB-infected trees and its level of spread varied significantly (*F* = 7.73, df = 28, *p* < 0.001; *F*= 16.90, df = 28, *p* < 0.001; *F* = 125.63, df = 4, *p* < 0.001) with treatment, sampling time and the treatment by sampling time interaction (Table 2). Significantly more citrus trees within untreated control plots were infected by HLB compared to HMO- treated plots over time (Table 2). The result demonstrates a significantly (*p* ≤ 0.001) higher percent of HLB prevalence on the untreated than the sprayed citrus plots over time (Table 2). The percent of HLB-infected citrus trees indicated typical expressions of the disease with leaf yellowing and patchy blotching built up gradually from 10% and 6% respectively for the untreated control and treated plots on 7 April 2012 to 38.7% and 15% respectively on 6 August 2012. Just 2.4% to 7.89% of these trees in control plots as compared with 0.25% to 0.51% in HMO-treated plots showed positive PCR outcomes. By 10 April 2013, 19.3% among the 56.2% of trees in control plots compared to 1.39% out of 18.60% in treated plots with expressions of slight blotchy and tiny leaves located upright displayed positive PCR outcomes. On 8 October 2014, 42.2% among the 80.2% of trees for control plots as compared with only 11.4% out of 40.3% of trees for HMO-treated plots exhibiting features of massive blotching and corky or enlarged vein on leaves together with several twig dieback indicated positive PCR probe. The present study showed that for the HMO-treated plots, the percentage of trees showed typical symptoms, and PCR positive results were significantly lower than the control plots. Field inspection on the scale of the characteristic visible indications for the affected trees can occasionally be confusing. These visual expressions could accredit to the nutrient insufficiency or other physiological dysfunctions. Thus, PCR is helpful for corroborating the presence of CLas bacterium in the HLB-affected trees. In China, citrus HLB generally develop rapidly in immature citrus grove; 50–70% of the citrus trees were affected prior to fruit-bearing [73,74]. The present study showed that HMO-sprayed plots greatly reduced the infection level to 11.40% as compared with 42.20% for the unsprayed plots after 4 years of planting. This reduction in HLB level is probably due to HMO interrupting the penetrating performance of CLas-prone *D. citri*; somehow, that influenced the CLas dissemination effectiveness and reduced feeding by adult psyllids and oviposition by adult females. This can assign the effect of HMO toward killing psyllid eggs, nymphs, and adults, and insecticidal application can bring about, importantly, adult fatality.

The reduction in the transmission of virus disease has been demonstrated by using specific petroleum oils which are recognized to decrease aphid settlement on plants [74,75]. Vandenveken [76] explained the possibility of the mineral oils to modify the charge, disrupting the adsorption of virus particles which would prevent the charge exchange in between virus particles and aphid mouthparts. Obviously, the vector control using HMO practised in this citrus orchard did reduce the vector *D. citri* numbers and the degree of HLB disease infection. The spread or transmission of HLB disease in the *D. citri*- invaded citrus grove seems to be correlated to increasingly soaring transmitter numbers and considerable inoculant stock. Furthermore, rapid spread of disease and sustained flow of flushing of young flush shoots was related [1,42,55]. Highest activity of ACP is mostly influenced by flushing rhythm of host plants and *Trioza erytreae* (Hemiptera: Triozidae) which is described to be highly infective when sucking or harboring on immature flush shoots of HLB-diseased trees [1]. Therefore, the vector was extremely attracted to the prolonged and sustained flow of young flush shoots throughout the flush rhythms of immature trees; this can partly be described as the fast transmission and dissemination of disease in the vector-affected trees inside the citrus grove. Derived from this finding, protection of healthy citrus from ACP invasion through the appearance and growth of a flush is essential. Presence of an array of volatiles in citrus flush shoot was observed to attract and might stimulate adult ACP feeding and probing on immature leaves more frequently and for prolonged periods than on mature leaves. Therefore, this may increase the risk of CLas transmission [20]. The appearance of flush would speed up disease development in an orchard. Hall and Moulton [77] reported that the number of psyllids, the degree of HLB infection, and the rate of dissemination are positively correlated. The wavelength of yellowish green >500 nm are strongly attractive to *D. citri* adults [78], and thus, the infected trees became attractive objects for boosting the percentage of disease-transmitting *D. citri*. In current research performed underneath planthouse conditions, it has been demonstrated that immature flush shoots become virulent in a period of 10–15 d following CLas injection [79], and the transmission rates of CLas are augmented by the appearance of immature flush shoots on citrus trees [80].

Koizumi et al. [81] reported that field-collected *D. citri* adult psyllids which were capable of transmitting HLB disease at a ratio of more than 41% come after feeding for 2 days on citrus flushes. DNA hybridization disclosed that the percentage of viruliferous *D. citri* adults observed in the affected citrus grove ranged from 5% to 39% respectively during May and September in Sarawak [82]. This report implied that it is not possible to eliminate the pathogen if there is a lack of effective vector and disease control programs for removing diseased trees. The contagious dispersal pattern encountered here for winged *D. citri* provided an explanation of the patchy spread of the HLB in the citrus orchard. Undoubtedly, ACP was answerable for the fast dissemination of HLB among trees over time inside the citrus grove. Application of selective insecticides [34,35,36,37] to rotate with petroleum oil sprays and to be supplemented by biological control agents such as *Tamarixia radiata* (Waterston) and *Diaphorencyrtus aligarhensis* (Shafee, Alam and Argarwal) as well as several ladybeetle species and Argentine ant *Linepithema humile* are advocated for development of an integrated pest and disease management strategy of *D. citri* [33,38,39,40,41,42,43,44]. This will definitely discourage and impede HLB disease transmission through rapid suffocation and prolonged insects’ injury process. According to Milosavljevic et al. [45], density-dependent reduction of ACP by *T. radiata* was identified as a significant mortality factor often exceeding 60% during periods of peak parasitoid activity and is required to maximize biological control of *D. citri*. Therefore, it is imperative to supplement with selective insecticides or HMO application at vital flushing durations which are possibly efficacious comparatively in inhibiting dissemination of disease and can request consistent surveillance of *D. citri* population in citrus orchards that it would be possible to use time management strategies such as trapping, regular monitoring, and insecticide applications. Stelinski [83] proposed that it might be useful to safeguard citrus trees from *D. citri* infestation by the installation and preservation of visual and physical barriers. *D. citri* management must consider wide-area collaboration, grove boundary monitoring and treatment, elimination of alternate-host habitat, and an understanding that immigrating adults may come from a short range within certain kilometers.

## 4. Conclusions

The outcomes of this research emphasize the significance of immature flush abundance within the dynamics of *D. citri* population with an outcome for management of the vector. The emergence of new flush cycle initiates a surge in adult numbers of *D. citri*, and a drop was noted while flush shoots reach maturity and reduced availability of young flush shoots in citrus orchards. Our results corroborate such findings by other authors who have also shown similar correlations between the cumulative temporal ACP populations and citrus immature flush patterns over time. The relative degree of aggregation (RDA) was higher in rainy months during the high population number of *D. citri* when adult emergence peaked and declined gradually as the population number decreased with the emigration and dispersal of adults.

The field experiment showed that horticultural mineral oils (HMOs) is effective against *D. citri* and can reduce HLB transmission in citrus orchards. Application of HMOs to rotate with selective insecticides and to be supplemented by biological control agents are advocated for development of integrated pest and disease management strategies of *D. citri* to reduce *D. citri* populations in orchards, to minimize HLB dissemination and to protect citrus trees from HLB infection. The major concern in controlling HLB is to avoid and/or minimize CLas-infected *D. citri* through uncontrollable fields nearby the commercial citrus farms.

## Figures and Tables

**Table 1:** Effect of horticultural mineral oil (HMO) on numbers and flush shoot infestation levels of *Diaphorina citri* during August 2012.

Treatment	Mean Number ^1^			Percent of Infestation ^2^		Total
	Adults	Eggs	Nymphs	Adults	Eggs	
HMO	3.79 ± 0.79	1.39 ± 0.09	2.66 ± 0.66	11.44 ± 0.14	8.99 ± 0.54	5.65 ± 4.04 ^A^
Control	7.80 ±1.47	4.30 ± 0.44	5.41 ± 0.20	35.75 ± 0.75	39.99 ± 1.89	18.65 ± 16.37 ^B^
Total mean	5.80 ± 2.44 ^a^	2.85 ± 1.62 ^b^	4.04 ± 1.56 ^b^	23.60 ± 13.32 ^c^	24.49 ± 17.02 ^c^	

^1^ Data √x + 0.5 transformed prior to ANOVA; ^2^ Data angular transformed prior to ANOVA; ^A,B^ (*F* = 1608.15, df = 1, *p* = 0.000); ^a,b,c^ (*F* = 352.11, df = 4, *p* = 0.000).

**Table 2:** Effect of horticultural mineral oil (HMO) on percentage of HLB-infected trees in a citrus orchard.

Tree	Treatment	Sampling Date					Total
		7 April 2012	6 August 2012	10 April 2013	20 March 2014	8 October 2014	
SP	Control	10.00 ± 2.00	38.70 ± 2.70	56.20 ± 1.20	62.30 ± 1.13	80.15 ± 1.23	49.47 ± 24.65 ^A^
	HMO	6.00 ± 2.00	15.00 ± 1.00	18.60 ± 1.68	25.20 ± 1.11	40.28 ± 1.60	21.02 ± 11.93 ^B^
DP	Control	2.41 ± 0.50	7.89 ± 0.81	19.19 ± 6.97	28.20 ± 12.61	42.20 ± 18.59	20.04 ± 14.76 ^a^
	HMO	0.25 ± 0.51	0.51 ± 0.58	1.39 ± 0.96	5.26 ± 2.50	11.43 ± 3.68	3.77 ± 4.41 ^b^
Total		8.00 ± 2.83 ^1^	26.85 ± 13.11 ^2^	37.40 ± 20.64 ^3^	43.75 ± 20.35 ^4^	60.22 ± 21.84 ^5^	

SP = symptomatic tree; DP = Diseased tree. ^A,B^ (*F* = 7.73, df = 28, *p* = 0.000); ^a,b^ (*F* = 16.90, df = 28, *p* = 0.000); ^1,2,3,4,5^ (*F* = 125.63, df = 4, *p* = 0.000).

## Data Availability

Not applicable.
